# Nursing Students’ Perceptions about Effective Pedagogy: Netnographic Analysis

**DOI:** 10.2196/27736

**Published:** 2021-06-22

**Authors:** Jennie C De Gagne, Paula D Koppel, Hyeyoung K Park, Allen Cadavero, Eunji Cho, Sharron Rushton, Sandra S Yamane, Kim Manturuk, Dukyoo Jung

**Affiliations:** 1 School of Nursing Duke University Durham, NC United States; 2 College of Nursing University of Massachusetts Amherst Amherst, MA United States; 3 School of Nursing Vanderbilt University Nashville, TN United States; 4 Department of Nursing Catawba College Salisbury, NC United States; 5 Duke Learning Innovation Duke University Durham, NC United States; 6 College of Nursing Ewha Womans University of Korea Seoul Republic of Korea

**Keywords:** discussion forums, faculty behaviors, health professions students, learning environment, learning experience, netnography, pedagogy

## Abstract

**Background:**

Effective pedagogy that encourages high standards of excellence and commitment to lifelong learning is essential in health professions education to prepare students for real-life challenges such as health disparities and global health issues. Creative learning and innovative teaching strategies empower students with high-quality, practical, real-world knowledge and meaningful skills to reach their potential as future health care providers.

**Objective:**

The aim of this study was to explore health profession students’ perceptions of whether their learning experiences were associated with good or bad pedagogy during asynchronous discussion forums. The further objective of the study was to identify how perceptions of the best and worst pedagogical practices reflected the students’ values, beliefs, and understanding about factors that made a pedagogy good during their learning history.

**Methods:**

A netnographic qualitative design was employed in this study. The data were collected on February 3, 2020 by exporting archived data from multiple sessions of a graduate-level nursing course offered between the fall 2016 and spring 2020 semesters at a large private university in the southeast region of the United States. Each student was a data unit. As an immersive data operation, field notes were taken by all research members. Data management and analysis were performed with NVivo 12.

**Results:**

A total of 634 posts were generated by 153 students identified in the dataset. Most of these students were female (88.9%). From the 97 categories identified, four themes emerged: (T) teacher presence built through relationship and communication, (E) environment conducive to affective and cognitive learning, (A) assessment and feedback processes that yield a growth mindset, and (M) mobilization of pedagogy through learner- and community-centeredness.

**Conclusions:**

The themes that emerged from our analysis confirm findings from previous studies and provide new insights. Our study highlights the value of technology as a tool for effective pedagogy. A resourceful teacher can use various communication techniques to develop meaningful connections between the learner and teacher. Styles of communication will vary according to the unique expectations and needs of learners with different learning preferences; however, the aim is to fully engage each learner, establish a rapport between and among students, and nurture an environment characterized by freedom of expression in which ideas flow freely. We suggest that future research continue to explore the influence of differing course formats and pedagogical modalities on student learning experiences.

## Introduction

### Background

Pedagogy is defined as the science and art of teaching practice, and is informed by complex learning theories and principles [1]. Effective pedagogy that nurtures high standards of excellence and commitment to lifelong learning is particularly meaningful for health professions education (HPE) to prepare students for real-life challenges such as health disparities and global health issues. Recognizing that their preparation as health care providers often places students in unfamiliar settings, HPE scholars [2,3] contend that it is important to leverage such sites to facilitate transformative learning and the motivation to grow. Accordingly, transformative learning theory has a broad application within HPE, and feminist pedagogy is similarly applicable given the rise of more inclusive and transformative learning environments to generate humanizing experiences for students [4].

### Transformative and Feminist Pedagogy

Transformative learning theory is grounded in Mezirow’s [5] belief that the value of educational programs relies on the perspectives of individuals, groups, and stakeholders in the evaluation process. The STAR (Sensitivity, Taking Action, and Reflection) framework synthesizes doctrines from transformative learning to support the changes in teaching strategies and curriculum in nursing education that are needed for the 21st century [2]. The STAR framework leverages the synergy between transformative learning and nursing education, highlighting a humanistic focus and holistic teaching strategies to cultivate empathy and compassion [2]. Feminist pedagogy similarly rejects the traditional teacher-student hierarchy, while encouraging students and teachers to use personal experiences as essential resources to evaluate perspectives critically and contemplate shifts in beliefs [6].

Feminist pedagogy is defined as “a theory about the teaching/learning process that guides our choice of classroom practices by providing criteria to evaluate specific educational strategies and techniques in terms of the desired course goals or outcomes” [7] (page 8). Feminist theorists suggest that teachers provide activities that develop critical thinking by tapping into the “disequilibrium” created by using a feminist teaching approach [8]. This process involves choosing content and assignments that allow students to examine, question, and create new knowledge, as well as encouraging them to write to learn rather than to demonstrate acquisition of knowledge. Teacher role modeling plays an important role in feminist pedagogy, allowing students to provide significant input into course development and ensuring that all students’ voices are heard in class discussions [8]. The teacher supports trust and sharing by creating a safe environment, and remains receptive to changing class activities or content to promote enhanced student perspective and reflective dialogue [9].

Experience, reflection, and change are at the heart of transformative and feminist pedagogies: both encourage students to process information acquired through personal experiences, values, feelings, and conditioned responses, and both emphasize a learning process guided by discourse, dialogue, and reflection [10,11]. Rooted in the social change movements of the late 1960s and early 1970s, feminist pedagogy focuses on raising consciousness and empowering vulnerable and oppressed groups [11,12]; thus, both the STAR framework and feminist teaching approaches aim to familiarize and engage nursing students with social justice issues [2].

Although many primary tenets of feminist pedagogy are already reflected in teaching practice [4], online learning formats pose challenges to several fundamental characteristics of the theory, such as the ability of teachers and students to cocreate the classroom experience when learning modules are prepared by the teacher in advance [6]. The theory of community inquiry [13] can contribute solutions for some of these challenges in online pedagogy and research.

### Community of Inquiry Framework

The community of inquiry (COI) framework emerged within a study as researchers sought ways to code and analyze computer-mediated communication such as asynchronous online discussion forums; however, this framework has also been used to support online pedagogy as the basis of cognitive, social, and teaching presence [13]. One of the most important components of online pedagogy is active engagement, and discussion forums are effective instructional strategies for fostering collaborative learning in varied domains [14]. Categories and indicators for each of the three elements of presence are sufficiently broad to be useful in analysis of transcripts but specific enough to be meaningful [13]. Because the evolution of this framework is in line with our project’s scope, we considered that it may provide a useful guide for exploring our text-based discussion forum data. Interestingly, the work of Garrison and colleagues [13] provides some of the first empirical evidence that written or text-based communication is generally better at producing high-order critical thinking, and that community and social context are important to achieve this more advanced level.

As suggested by transformative and feminist pedagogies and the COI framework, the role of the educator (whether in person or at a distance) is that of a colearner as well as a facilitator who recognizes learners’ objectives and goals, and creates a safe forum for discussion and reflection. The educator maintains control of the learning setting but is not controlling of the learning process. Classroom strategies focus on empowerment by providing class members with opportunities to develop goals and objectives, develop autonomy, enhance decision-making, and boost/reinforce their self-esteem [12]. By reimagining the classroom as a shared learning community [12], educators facilitate the achievement of students’ goals, propel them toward autonomy [10], and empower them to create and advocate for positive change as they assume their professional roles [2].

### Research Aim

The aim of this study was to explore health profession students’ perceptions of whether their learning experiences were associated with good or bad pedagogy during asynchronous discussion forums. A further objective of the study was to identify how perceptions of the best and worst pedagogical practices reflect the students’ values, beliefs, and understanding about factors that made a pedagogy good during their learning history.

## Methods

### Netnography

As the context of the data collected for this study was an online learning course, netnography was an ideal methodology. Netnography developed as a subgroup of the ethnographic research tradition and is specifically designed to examine the practice of distinct social interactions [15]. Described by Kozinets, its creator, as a way to analyze “technocultural contexts” where culture and technology utilization meet [16], netnography always focuses on social media and technoculture; includes the immersion of the researcher; and uses impressions to inform cultural understanding of the nexus where culture, technology, and society intersect [15,16]. Netnography examines any phenomenon within this domain that has become a key component of our collective experience as humans; it distinguishes itself as a method designed to illuminate the emotional story and meaning of online life [16]. Netnography uses the following steps: (1) planning and including a cultural entrée, (2) collecting data, (3) performing ethically based research, (4) interpreting data, and (5) determining a data presentation plan [17]. This methodology requires that the investigators be fully immersed in the online community to gather data through participant observation [18]. Investigators may also conduct interviews and gather archival data, field notes, and other forms of data [17]. Additionally, investigators use reflection to better understand the community [18].

### Study Design, Participants, and Setting

A netnographic qualitative design was used to explore the views and experiences of students who participated in online forums in a graduate-level nursing course that teaches themes of adult learning, learning styles, student engagement, domains of learning, teaching strategies, and/or methods of integrating technology into nursing education. Enrollment size ranges from 10 to 35 students; however, group dynamics and interactions among members are unlikely to be affected by differences in enrollment because students work in small groups of 4 to 5 in a discussion forum. The forum presents an opportunity for students to share their ideas and personal perspectives on each week’s course topics thoughtfully. During the course, students are expected to write an initial post in response to a question posted for the week and to respond to posts from at least two peers. The data for this study included the initial discussion forum posts and peer-response posts during the first or second week of the course. This project was reviewed and declared exempt by the Duke University Institution Review Board (Pro00104522).

### Data Collection Procedures

The data for this study were collected on February 3, 2020 by exporting archived data from sessions of the nursing course run between the fall 2016 and spring 2020 semesters. In each session, students were given the discussion forum prompt shown in Textbox 1. This prompt asked them to describe their best and worst learning experiences and to reflect on how these experiences were related to what they were learning in the course.

The analysis file used in this study included the original forum prompt and all of the nested replies to that prompt. The study data were deidentified, cleaned, and placed into Microsoft Excel 365 software and exported to NVivo qualitative data analysis software (QSR International Pty Ltd) prior to analysis.

Although netnography typically requires researchers to immerse themselves in the online community during data collection, our study collected investigative data from the course discussion forum retrospectively; thus, field notes were taken by all research members as an immersive data operation. Among the nine researchers of this study, six are nurse educators. In addition, our research team included the professor, teaching assistants (TAs), and previous students of the course. This immersive data operation was deliberatively performed to serve as a “reflective, catalytic, and analytic guide” [19] for the data analysis by teachers and observers of the students’ online discussion forum.

**Discussion forum prompt.**

Think about the best learning experience you’ve had. It can be any kind of course and taken at any time in your learning history. Now ask yourself WHY this was such a good learning experience. What was it about the focus of the course, what the teacher did, what you were expected to do, the course assignments, and so on that made this such a positive experience? Reflect on what you’ve heard and read about principles and theories of learning and discuss how your very positive experience does or does not confirm what the theorists say about how people learn, good principles of education, the factors that influence learning, etc.

Now think about the worst learning experience you’ve had. Think about what made it so bad, which principles of learning were “violated,” and what could have made the learning experience better for you.

In both cases, you should feel free to describe the course (eg, the leadership course in my undergraduate nursing program), when you took it (eg, this was the last semester before we were to graduate), and anything else that may help the rest of us understand the context (eg, there were 60 students in this course, and we had been together in many courses before taking this one; the teacher was new to the school but not new to teaching). Connect your thinking and experience with what you’ve read and consider whether your experiences were unique or whether they were similar to those of other students enrolled in that same course. In all discussions, please do not mention names of professors or schools.

### Data Analysis

Data management and analysis were performed with NVivo 12. Each student is a data unit. Our analysis primarily focused on the initial post by each student, although follow-up posts in response to other students were also included in the analysis as they reflected students’ learning experiences. An inductive approach was used to code the data. The data units were divided among the team members and in vivo coding was generated to ensure that the first-level coding was grounded in the participants’ experience [20]. Field notes in the form of reflections/memos were also created iteratively as authors read and coded the data, and were included as part of the analysis. Each member read the discussion thread several times to get a sense of the whole and generated field notes as a format of free writing. This included reflective memos to capture both insights and bracket personal perceptions that might have influenced the analysis, making the team members’ personal beliefs and experiences transparent [20].

During the coding process, first-level coders (AC, EC, SY, SR, and DJ) generated a total of 1019 in vivo codes. Three team members (JD, PK, and HP) completed second-level coding by exploring patterns and relationships among the in vivo codes. Codes and categories generated by coders were reviewed by two research members (PK, HP), and each step of data analysis was discussed during the regular research team meetings. This resulted in the development of 97 categories (50 positive aspects, 33 negative aspects, 14 neutral aspects). The categories were reviewed and discussed during team meetings for consensus on themes. The categorization and theme generation required an iterative process to ensure the incorporation of as many of the participants’ experiences as possible into the final themes.

### Rigor/Trustworthiness

The research team members met regularly to discuss and refine all levels of the data analysis process. The data analysis process and personal impressions of the data were carefully and consistently recorded in analytical memos [20], which included all major analytical decisions (ie, code revisions, recoded data, data organization, and labeling processes) as well as insights and relationships observed. This process resulted in a detailed audit trail to help promote transparency [21]. Finally, the research team selected categories that represented a wide range of ideas and topics present in the data. Exemplar quotes were selected for each major theme and subthemes to document evidence thoroughly for the study’s findings [22]. These processes allow readers to determine the application of our results to their own contexts and more easily reproduce the study [23].

## Results

### Sample Characteristics

The total number of posts was 634, generated by 153 students identified in the dataset. Most of these students were female. Many of the students were enrolled in the Doctor of Nursing Practice and Master of Science in Nursing degree programs. Others were students from outside the nursing discipline. Details of the sample can be found in Table 1.

**Table 1:** Descriptions of study participants (N=153).

Characteristic			Participants, n (%)
**Gender**			
	Female	136 (88.9)	
	Male	17 (11.1)	
**Participation per semester**			
	Fall 2016	36 (23.5)	
	Spring 2017	11 (7.2)	
	Fall 2017	9 (5.9)	
	Spring 2018	16 (10.5)	
	Spring 2019	33 (21.6)	
	Summer 2019	13 (8.5)	
	Fall 2019	22 (14.4)	
	Spring 2020^a^	13 (8.5)	
**Degree**			
	ABSN^b^	4 (2.6)	
	BSN^c^ to DNP^d^	11 (7.2)	
	DNP	63 (41.2)	
	MSN^e^	61 (39.9)	
	PhD^f^	6 (3.9)	
	Other	8 (5.2)	

^a^Data were collected prior to the COVID-19 pandemic in the United States.

^b^ABSN: Accelerated Bachelor of Science in Nursing.

^c^BSN: Bachelor of Science in Nursing.

^d^DNP: Doctor of Nursing Practice.

^e^MSN: Master of Science in Nursing.

^f^PhD: Doctor of Philosophy in Nursing.

### Overview of Themes

**Figure 1:**
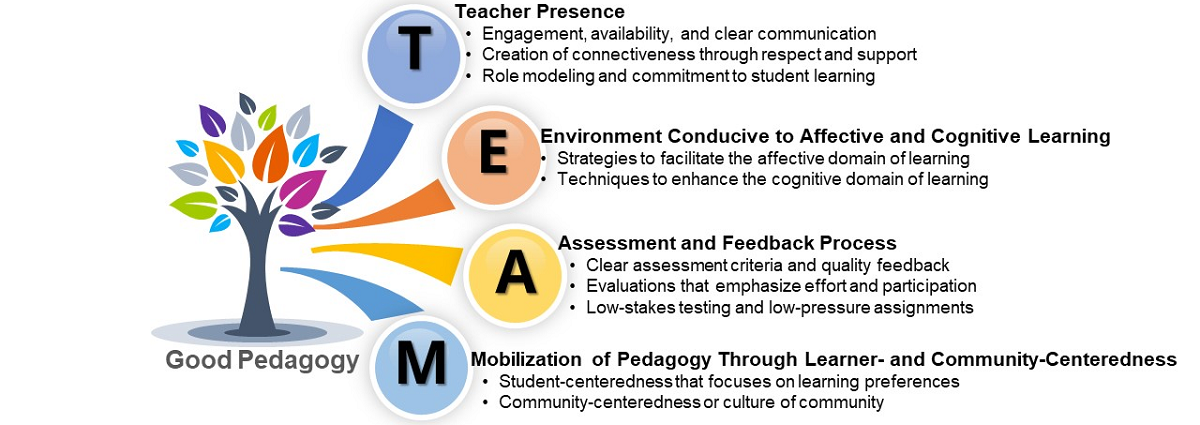
Overview of the key themes and their subthemes.

From the 97 categories identified, four themes emerged: (1) teacher presence built through relationship and communication, (2) environment conducive to affective and cognitive learning, (3) assessment and feedback processes that yield a growth mindset, and (4) mobilization of pedagogy through learner- and community-centeredness. We created an acronym (ie, T.E.A.M.) to help us remember these themes, depicted in Figure 1 as the principal findings of this study.

### T: Teacher Presence

#### Description of Main Theme

Students described their learning experiences as positive when the teacher was connected and actively involved in the learning environment. By contrast, negative learning experiences were associated with disengagement between teachers and students as well as with lack of teacher passion or empathy. When their teachers were perceived to be apathetic, defensive, indifferent, insecure, and difficult to access, students negatively evaluated their learning experiences. Students’ perceptions that they were not receiving needed support or respect from teachers interfered with their learning efficiency.

#### Engagement, Availability, and Clear Communication

Teachers were described positively when they were perceived as being eager to communicate and cocreate an active learning environment with students, or as truly caring about students and their learning. Vigorous and receptive teachers were considered approachable, open to communication with students, and able to deliver clear instructions and guidelines. One student explained, “[my teacher] made it clear at the beginning of the class what was expected from the class and how she was going to assist the class to navigate the course.” On the other side, lack of clarity was a source of frustration for the students. For example, one student said, “The teacher was so awful at explaining things that I walked out of that first lecture feeling more confused about things I had previously understood.”

#### Creation of Connectiveness Through Respect and Support

Teachers were identified as having provided positive learning experiences if they had created close and strong connections with students, often from the start of the semester: “I find that when professors introduce themselves and students do, that I connect more. It begins a relationship that can make learning more interactive.” Students who perceived that their teachers were willing to care and connect with them expressed that they felt supported and respected as members of the class and as human beings. A sense of participating in a humanistic teacher-student relationship stimulated motivation to learn and engage in the classroom. As one student noted, “The contact between the professor and the students was not only motivating, but truly enhanced the learning experience.” Students perceived teachers’ prompt responses, constructive criticism, and enthusiastic support as indications of a full effort to guide them to their highest potential. One student described their teacher as “a coach, counselor, cheerleader, and mentor,” expressing their perception that the teacher not only conveyed knowledge to students but also treated them as autonomous agents, guiding them with affection.

#### Role Modeling and Commitment to Student Learning

Students highly valued teachers’ devotion, time, effort, and professional expertise. Teachers with excellent expertise and profound knowledge in their fields were perceived as positive role models who were well-prepared and trustworthy. According to one student:

Teacher factors that impacted the learning experience included her high level of competence within nursing, her many shared experiences which were relevant to course material, very high level of motivation, and a positive personality that she brought to class.

### E: Environment Conducive to Affective and Cognitive Learning

#### Description of Main Theme

Students’ posts described qualities of the learning environment that supported or inhibited their learning. Students discussed cognitive learning, especially in relation to supportive teaching strategies, and frequently described experiences and characteristics of educators that promoted aspects of affective learning such as self-awareness, self-confidence, and values consistent with nursing behavior.

#### Strategies to Facilitate the Affective Domain of Learning

Students described a variety of strategies that instructors used to develop and foster confidence, motivation, and professional growth, thereby creating an environment that facilitated learning in the affective domain. Motivating students was identified as an important goal, but creating motivation appeared to require a holistic approach to pedagogy. For example, students expressed that high expectations from the teacher, when combined with professionalism and respect for students, created an atmosphere that ignited constant learning. As one student stated, the teacher taught the students “as adult learners, and it was incredibly refreshing. She respected us, set high expectations, maintained professionalism, and was a skilled leader.” Challenge combined with positive reinforcement was especially appreciated by a student who stated, “I need validation from professors. I need to know I am going in the right direction.”

Students not only expressed that their best experiences involved courses with high expectations but also described easy classes as their worst experiences. For example, one student noted that “even though most of the students in her class obtained good grades, it did not feel like we earned them because she did not challenge her students and spoon-fed us the answers.” Self-awareness or self-reflection was noted as a strategy that also facilitated learning in the affective domain. As one student noted, “[The teacher] helped the nursing students explore their possibility and build their beliefs of being nurses.”

#### Techniques to Enhance the Cognitive Domain of Learning

Positive techniques perceived as facilitating learning in the cognitive domain included promoting a spirit of inquiry through questioning and goal-setting. Creative activities were also appreciated, as one student explained: “[The teacher] kept learning interesting by introducing new opportunities to meet objectives in unconventional ways.” On the other side, students did not perceive all strategies as contributing to positive learning experiences. For example, rote memorization by faculty was often described as unhelpful. One student emphasized the importance of making connections beyond memorization of the material, stating that “memorization can be a great way to efficiently get a good grade on a test, but much of it is eventually lost since it is often without meaning.”

### A: Assessment and Feedback Process

#### Description of Main Theme

Assessment and feedback were frequently described in the posts along with comments on characteristics that enhanced and inhibited students’ learning experience. This included the type, frequency, and focus of evaluations as well as the manner in which feedback was delivered.

#### Clear Assessment Criteria and Quality Feedback

Students reported that a higher level of learning was achieved when they were provided with clear instructions and expectations for assignments and deadlines. Lack of organization and structure as well as grading and assessments that did not contain material covered in class or other resources were identified as contributing to negative experiences, as illustrated in one student’s reflection:

The professor would jump from topic to topic, would skip key concepts, and was not very organized. The tests often contained material that was not covered in class or within the assigned readings, and she often misplaced our assignments.

Other negative experiences were associated with tests that did not assess a real understanding of concepts from the material. Providing meaningful feedback on tests and papers, both negative and positive, was identified as an important way to improve student performance. As one student stated, “I learn and grow best with a healthy amount of constructive criticism.” The use of verbal feedback created a lasting impression and invoked a sense of pride in students. As one student expressed:

The input was not only in the form of a grade but also verbal. I may not remember the words said but can remember the sense of pride I felt and the body language of the teacher communicating my success.

#### Evaluations That Emphasize Effort and Participation

A strong desire was expressed for a shift to assessments and grading focused on learning in lieu of letter grades. Students reported that a focus on learning made them feel more invested in learning and enhanced their ability to gain knowledge. One student shared that “when I’m not focused on the letter grade, I find myself more invested in the learning experience as a whole and leave with a whole new set of knowledge.” Examples provided included an emphasis on assessment of participation, and evaluations that described how students exhibited a desire to learn. Another student noted that tracking participation in class increased engagement and eventually led to a better learning experience.

#### Low-Stakes Testing and Low-Pressure Assignments

Several comments illustrated that low-stakes testing was a valuable tool for learning. For example, they expressed that noncumulative exams and incremental assignments relieved pressure compared to higher-stakes testing and evaluation. One student explained, “There are three exams. And it is not cumulative, which means a lot of relief at the end of the semester.” Another preferred approach was the use of short-answer responses on quizzes about the application of concepts learned in class. Homework assignments that encouraged students to examine the material presented in lectures in greater depth were described as facilitating understanding.

### M: Mobilization of Pedagogy Through Learner- and Community-Centeredness

#### Description of Main Theme

The fourth element of good pedagogy was learner and community centeredness. One student described such an approach as evidenced by “an excellent teacher who is warm and accessible, respects our options and ideas, creates a sense of community and belonging.” Students emphasized the importance of creating a safe and nurturing learning environment.

#### Student-Centeredness That Focuses on Learning Preferences

Students made personal connections with course content that teachers illuminated with their past experiences and existing knowledge. Unfortunately, not all student experiences were positive. Teachers who were perceived as having ignored individual learning styles or overemphasized one teaching strategy were described as having disenfranchised the adult learner. One student reported, “Different styles of learning were not taken into account, and the large, bleak classroom and chalkboards were unstimulating.” Interestingly, students often reframed such negative experiences as ways of reassessing their learning needs or as teaching moments. For example, one student posted that “the bad experience certainly showed me what not to do, how not to behave, and what my future students will not want me to do.”

#### Community-Centeredness or Culture of Community

Teachers were highly esteemed by students when they actively engaged the learner through dynamic discussions, and valued group members’ ideas and opinions. Students expressed that they felt safe when encouraged “to express their feelings and learn to respect and listen to others.” When teachers fostered this type of open collaboration, students felt that a community of practice developed between the learner and teacher, promoting a culture of inquiry. On the other side, students expressed that teachers who used confrontational tactics, including public correction and shaming, disengaged the learner and broke the bond of trust and community. One student lamented, “I remember nothing from his class except my feeling of fear and sadness for my friends that were humiliated by this teacher.” 

## Discussion

### Reflections From the Research Team

Using the netnographic approach, this study analyzed asynchronous discussion forum posts by health profession students describing their best and worst learning experiences in an effort to understand their perceptions of what constituted or contributed to good pedagogy. Before addressing this specific aim, the reader is referred to Multimedia Appendix 1, in which each author has provided a brief reflection to describe their social identity and relevant experiences related to the study findings.

As is true of any qualitative study, our research process was undoubtedly influenced by the beliefs, values, experiences, and perspectives of the members of our research team, starting with the questions we selected to investigate. Although we analyzed data retrospectively, many of us had roles in the course where data were collected. Our research team was made up of an eclectic group of teachers, former students from the course, and researchers with various titles and roles within the academic profession. Reflective memos were used to identify our prior beliefs and values, and this exercise provided opportunities for bracketing the influences of our perspectives and made them transparent [21]. To strengthen our collective analysis of the data, we had multiple research team meetings in which we shared our personal worldviews and perspectives on good pedagogy as related to the data overall. This process is essential in netnography, a methodology that encourages participant observation with investigators immersed in an online community [18]; it helped us to explore our individual perspectives and consider the meaning of our experiences from multiple viewpoints. For example, one team member described their experience of reading student writing from two perspectives: first as a student in the course and then as an educator (a TA) in the course the following semester. This sharing of experiences during team meetings nourished team members’ reflections and increased the richness of our perspectives as researchers.

All of the researchers involved in this study have been students (most are either current students or have recently graduated), and all have been teachers or TAs. These experiences influenced our reading of the data in that we recognized the challenges involved in meeting individualized and diverse student needs within the constraints of our teaching environments. Acknowledging our prior experiences at the onset of the project was both a strength and a limitation; we purposely chose to start the analysis using in vivo codes to have the best chance of keeping the data grounded in the participants’ experiences.

### Student Perceptions of Good Pedagogy

Our findings confirm that nursing students consider a positive teacher presence and a strong teacher-student connection to be key elements of good pedagogy. Students’ descriptions showed that they perceived a transfer of knowledge alone to be insufficient for effective learning as they needed to feel motivated, inspired, and respected as human beings by the teacher’s presence. Our findings support several studies that have reported that humanistic connections and relationships with teachers can lead students to achieve positive learning outcomes and professional socialization [24-26]. Bergum [24] used the term “relational pedagogy” to highlight the importance of a teacher listening to students’ thoughts and responses, creating connections with students and the world, and inspiring students while being inspired by students. Furthermore, the inherent values of the teacher-student connection (eg, trust, respect, reciprocity, and recognition) can transform students’ perceptions and perspectives, creating a “place of possibility” that allows students to discover their personal and professional potential, and to achieve self-transcendence [26]. In a study of preservice teachers’ experience of learning a humanizing pedagogy, emotional bonding and positive relationships with students were reported as catalysts to address educational issues with care, trust, and respect [27]. These findings suggest that human relationships, connections, and respect between teachers and students are not optional but indispensable for a thriving learning environment.

By contrast, there have been discussions about maintaining a proper distance between teachers and students. Chory and Offstein [28] questioned the extent to which the personal, emotional, and professional nature of human interaction should be attempted in learning domains, where a caring relationship between the faculty and students is essential. Molloy and Bearman [29] discussed “intellectual candor” in HPE and questioned the extent to which teachers can openly show vulnerability while remaining credible. Admittedly, criteria for meaningful connections between faculty and students are ambiguous and complex, and faculty-student relationships can become overly intimate and personal unintentionally [28]. To protect faculty, educational institutions, and students in particular, teachers should establish mutually desirable and healthy relationships with students through constant self-reflection and close discussion with colleagues, mentors, and administrators [28]. Further research of teacher and student perspectives is required to establish concrete guidelines for professional teacher-student relationships.

Our study supports the notion that teachers need to consider how to create a supportive learning environment given its impact on learning outcomes [30]. The creation of a learning environment that supports affective learning was highlighted as an important pedagogical strategy for the students. To deliver high-quality patient care, health profession students must learn to apply affective domain skills such as ethics, critical thinking, and judgment to clinical situations [31]. The literature identifies that reflection is an important strategy for affective learning, which is consistent with our findings [32,33], and is also a tool to facilitate active learning [32]. In addition to self-reflection, educators should consider incorporating strategies such as think-pair-share, role playing, and simulation to strengthen the affective learning domain [30], as well as activities such as portfolios, volunteering, and learning contracts [31].

The findings of our study expand on previous literature suggesting that the learning environment, and specifically the educator, can have an impact on student motivation [34]. Kember et al’s [34] motivational teaching and learning environment framework describes findings similar to all four themes outlined in this paper. Although all eight elements of Kember’s model were evident in our data, those that align most closely with our themes include close teacher-student relationships, teaching for understanding, assessment of learning activities, and sense of belonging between classmates [34]. Similar to the findings of Kember and colleagues, some students in our dataset identified student-teacher rapport, a sense of community, teaching strategies that facilitated cognitive and affective learning, and the importance of feedback as elements that fostered their motivation.

Our findings also suggest that students value being adequately challenged by coursework, which is consistent with recommendations for medical educators based on a social cognitive model [35] and self-determination theory [36]. These recommendations suggest that motivation to learn is an interaction of internal and external factors, and exploring ways of stimulating internal motivation [35,36]. In addition to using activities that provide challenge, other recommendations that were supported by our findings include promoting student-centered learning, effective feedback, and a sense of connectedness with the teacher and community.

An important form of interaction between students and instructors is assessment and feedback. Assessment and evaluation in nursing education are essential to the learning process [37]. Assessment is the process of gathering information about students, courses, educational programs, and policies. Assessment provides educators with information to make decisions about student performance, proficiency, and learning. It also produces feedback for students to develop their knowledge and skills, and to evaluate whether they have reached learning goals and outcomes [37]. In our study, students reported a higher level of learning when the instructor/facilitator provided clear instructions and expectations regarding assignments and deadlines, and feedback that improved student performance and instilled a sense of pride. Our findings support that assessment and its communication are key elements in successful pedagogy and best practices for implementation.

The students in our study described having good pedagogical experiences when learner and community building was mobilized. Humans are social creatures and need interaction to create a learning environment that actively involves students in the learning process [38]. Online education is becoming standard in higher education. As of the fall of 2018, over 35% of undergraduate students and 40% of graduate students were enrolled in at least one online course [39]. Student perspectives on the COI model in our study are consistent with assertions that active engagement and effective communication are essential in online learning communities [14] and provide an opportunity to socialize and feel more connected [38,40]. The COVID-19 pandemic has stressed the importance of being able to reach students remotely, and has confirmed both the benefits and the challenges of online education. The discussion board has emerged as a crucial methodology for instructors to provide interactive, active, and collaborative learning [14,38,40]. Some even argue that the best teaching occurs in asynchronous online discussion forums [38]. Modeling good online practice, summarizing posts, and responding to student posts consistently and often have been shown to encourage critical thinking [41] and higher-level learning [40]. As in transformative learning and feminist pedagogy, an interactive discourse is essential to mobilizing critical thinking and cocreating new knowledge in the COI framework. The students in our study described their learning experiences as positive when their teachers fostered a sense of community and meaningful collaboration while accommodating individual learning styles and preferences.

We also discovered that individual students responded to or perceived specific teaching strategies and teacher characteristics differently. Interestingly, some students perceived high expectations as indicative of being respected as learners, whereas others wrote that their worst experience was related to high expectations. High expectations combined with respect or rapport, or with a supportive human relationship were more often viewed as components of good pedagogy. Despite some conflicting views, clear and strong themes emerged from the data demonstrating the importance of professionalism, caring, respectful student-teacher engagement, clear communication, and timely and thoughtful feedback for creating an effective learning community characterized by good pedagogy.

### Limitations

This study has several limitations. First, the data analysis and its interpretation depend on the researchers’ skills, assumptions, and experience; therefore, we took great care to maintain rigor during all levels of the coding, and we reflected on and shared our personal worldviews at the onset. Additionally, we involved a large team of researchers with diverse cultural backgrounds and different levels of teaching experience in the analysis and interpretation to ensure a multiplicity of perspectives. Second, our analysis depends solely on archived data, and we were unable to carry out member-checking of the data. We cannot know whether our interpretations of the sentiments expressed by the students in their writing accurately represent what they were experiencing or feeling at the time of posting. The nature of textual data in a netnographic study [17] also limits the ability to detect participants’ emotions or states of mind in the asynchronous online forum. Finally, the students in this study came solely from a private university in the southeastern United States. There were differences in age, nationality, race/ethnicity, and level of education among the students who participated in this study; however, there was homogeneity in that some study participants were enrolled in the same educational institution. Although this homogeneity may hinder generalization of the results to populations in other countries or areas, we did not ask students to limit their reflections only to educational experiences at their current university; therefore, it is likely that the range of experiences we coded, both positive and negative, represent experiences from many different learning environments.

### Future Studies

Although this analysis identifies several key aspects of high-quality education experiences, there remain some unanswered questions that can be addressed in future research. First, we note the tension that can exist between student wants and the pragmatic realities of teaching. For example, our analysis found that students want to receive frequent and detailed feedback on their work throughout a learning experience; however, instructors will find this difficult to accomplish when teaching large classes. Such tensions resonated with the research team, many of whom had recent related experiences as both instructors and students. Future research should examine different models for integrating or balancing the needs of students and instructors in larger classes.

Second, we note the importance of finding the right balance between the amount of work assigned and its level of difficulty. Students’ descriptions of poor learning experiences included those in which the work was too easy as well as too difficult. The same pattern emerged regarding the amount of work assigned. Good pedagogies provide a challenging yet manageable amount of work. Future research is needed to identify strategies that instructors can use to establish the right balance in a course. We hypothesize that this balance will vary based on course type, level, student population, and teacher characteristics, as well as pedagogical strategies and philosophies of education.

Third, future research should further elaborate the relationships between critical components of good pedagogy. For example, the role of student motivation in mediating learning experiences could be explored. Our data showed that teacher professionalism, adequate design of the learning environment, supportive challenges, and sense of connection with the teacher inspired and motivated students to embrace learning for personal and professional enrichment rather than as a means of obtaining a high grade. Although our study cannot verify a causal relationship, Keller [42] highlighted the critical role of motivation in learning and proposed the ARCS-V (attention, relevance, confidence, satisfaction, and volition) model, which can provide practical strategies to build and sustain student motivation. Future research can be performed to develop a conceptual framework of good pedagogy and explore the specific role of each component identified in this study, including motivation.

Finally, we did not ask the students to identify whether the positive and negative learning experiences they described occurred online, in person, or in hybrid courses. It is probable that different course formats and modalities should emphasize different elements of good pedagogy. For example, community building may be more critical in online classes in which students have no extracurricular engagements, whereas in campus-based courses that make greater demands on students’ time, managing the workload may be more critical. Interestingly, a recent study by Jezuit et al [43] surveyed nursing students in an online graduate program about faculty caring in their online program. Their results appear strikingly similar to ours. They identified four themes: (1) demonstrates engagement (ie, responsive, available, accessible); (2) facilitates learning (ie, timely, personalized feedback), which is similar to our assessment theme; (3) challenges students (ie, shares expertise, poses critical intellectual questions), which is similar to our environment for affective and cognitive learning theme; and (4) encourages students (ie, expresses empathy and compassion, provides praise, reaches out), which is similar to our teacher presence and mobilizing learner- and community-centered approach. We suggest that future research continue to explore how differences in course formats and modalities influence good pedagogy.

### Conclusions

We explored perceptions of good pedagogy by analyzing students’ descriptions of their best and worst learning experiences. The themes that emerged from our analysis confirm findings from previous studies and provide new insights. The essence of pedagogy must be high-quality, practical, real-world knowledge and skills that empower students to reach their potential. Good pedagogy is more than an instructional platform; students can have good and bad learning experiences on any platform. Instructional platforms are tools that an effective teacher uses skillfully to encourage maximum achievement. When utilized by the unskilled or inattentive teacher, however, the same tools can yield disappointingly different results. Virtual platforms pose unique challenges to observing the theoretical tenets of transformational learning and feminist pedagogy. Our analysis highlights the critical need to view technology as a tool in the service of pedagogy. Technology can facilitate the implementation of student-centered teaching approaches but cannot create them. Indeed, the COI framework embraces the feminist pedagogy by breaking down barriers between the teacher and learner, and creating the community necessary for higher-level learning. A resourceful teacher will embrace various communication techniques to develop meaningful teacher-learner connections. Styles of communication will vary as each unique group of learners presents different expectations and learning preferences. The aim of all should be to engage each learner fully by establishing a rapport and environment that allows the free flow of ideas and expression.

## Appendix

Multimedia Appendix 1 Reflective notes from each author.

## Abbreviations

COI: community of inquiry
HPE: health professions education
STAR: sensitivity, taking action, and reflection
TA: teaching assistant
