# Supplementary material for: Nursing Students’ Perceptions about Effective Pedagogy: Netnographic Analysis
Source: JMIR Med Educ. 2021 Jun 22;7(2):e27736. doi: 10.2196/27736 (PMC8277346; doi:10.2196/27736)
Supplement: Multimedia Appendix 1 [file mededu_v7i2e27736_app1.pdf]

Multimedia Appendix 1. Reflective notes from each author.

AC is a white, middle aged male nurse with over 10 years teaching experience. Although he has two graduate degrees in nursing education (MSN/PhD), he himself struggled academically throughout his primary and secondary education. Many sensational role models and mentors have lifted and often at times carried him during his undergraduate years. Coming from an Italian family that valued hard work, he was the first to attend and finish college despite many challenging obstacles. His own academic experiences have shaped his overall teaching philosophy to include learner centeredness and valuing learners at all levels. His strong belief in the adult learning theory concepts: empowerment, partnering, and caring were all behaviors he encountered throughout the academic journey.

DJ is a middle-aged Asian woman who completed her PhD in the United States. Upon completion, she returned to her home county and started teaching at one of the high-ranking universities in South Korea. As a visiting scholar at a university where this course was offered, DJ had the opportunity to audit the course and engage in the research project. DJ noted that it was surprising to her to find that students had high standards on teaching practice. Even though DJ has taught for more than 10 years, she rarely thought about good pedagogy in that when she taught the class, she mainly focused on delivering the course content. However, when DJ participated in this study, she realized that teaching was like an orchestration of many elements that emerged from this study. The discrepancy between DJ's past teaching practice and what she has learned from this research makes her anxious to begin using these new insights to improve her teaching.

EC is an Asian woman who had worked as a graduate TA and a teacher for BSN students for more than 4 years in South Korea. EC took this course first as a 4th-year doctoral student, and then she took it again as a TA in her final year of the doctoral program. While her identity was shifting from a long-time student to a novice educator, participating in the same class twice made her aware of the confusion and difficulties that teachers often face, just as she did when she taught BSN students. When participating in the discussion forum as a student, she also wrote that her best teachers were able to develop humanistic relationships with students. It seemed obvious that most students loved humane teachers with passion, professionalism, and character. However, when joining the discussion forum as a TA and reviewing these demands from the instructor's perspective, several questions arose: to what extent can human connection be tolerated, how can this connection be formed, to what extent is it essential, and to what extent is it just an additional bonus. EC noted that the principles of general human relationships may not be fully applicable to the teacher-student relationship because the instructors play their persona as a teacher when interacting with students. She continues to search for ways to develop mutually beneficial, healthy relationships between teachers and students.

HKP is an assistant professor in an academic institution in the United States. She completed her undergraduate and graduate education in South Korea and completed her PhD in the United States. When this research project was started, she was transitioning from student life to a role as an educator. During the research period, she was very interested in learning about students' perspectives on good pedagogy and began to recognize discrepancies between educators' and learners' expectations in relationship to time, effort, and the teaching content for courses as these

were a source of stress in her new educator role. HKP also experienced cultural differences that affect the pedagogical approach and the expectations for teachers and students. She still related to many of the student's posts, but now as a teacher also felt the high expectations of students and its associated burden.

JCD is a middle-aged woman who has taught this course since 2015 every semester. She obtained her nursing degree in South Korea in the 1980s, moved to the United States in the 1990s, and obtained four graduate degrees including a PhD in Education while working as a nurse, nursing director, clinical instructor, or professor. As a lifelong learner, JCD believes that investing in education is the most powerful and the best investment one can make. She has a strong opinion about learners taking responsibility for actively promoting continuous social change by building democracy and civility in the learning community. JCD advocates a self-directed and self-determined learning philosophy that encourages students' personal and professional growth and collaborative planning and action-taking. As an educator valuing and practicing constructivism learning theory, JCD strives to create an engaging student-centered learning environment where the students can know how to find and utilize instructional materials to meet their own learning needs, abilities, and interests. As such, an interpretation of the data in this project has likely been affected by her own personal and academic backgrounds, teaching philosophy, and reactions from the students she encountered. Honoring students' diverse perspectives and learning experiences, JCD has facilitated this project as a way of reflecting our own pedagogical practice and philosophies to continue to improve as educators.

KM is a middle-aged white woman who completed her PhD in her late 30's after leaving the workforce for several years to raise a family. As a non-traditional student who balanced graduate studies with family responsibilities, she developed a deep commitment to student-centered pedagogies and flexible learning experiences. KM has taught undergraduate-level sociology courses to very diverse populations ranging from students at an elite university to incarcerated individuals. As a result, she is particularly aware of the need to consider diverse and heterogenous student needs when teaching. In the content of this research, KM was particularly struck by the fact that good learning experiences often involved both cognitive and affective learning, outcomes she hopes to better foster in her teaching experiences in the future.

PDK, a white, middle-aged PhD student in the United States, experienced the course where data was collected as a student and TA. PDK is also a holistic health coach and teaches adult learners in both community settings and healthcare organizations. Her expertise in holistic approaches to care reflect a belief in the healing power of nurse-patient and teacher-student relationships. Returning to her own discussion post as a student reminded her that the data collected in the study includes an important pretense to consider when evaluating the findings. The discussion post directions required students to connect their personal experiences with the teaching principles and theories presented in the course. PDK recalled that for this assignment she immediately recollected her best and worst experiences. To develop of her discussion post, she reviewed the teaching-learning principles presented in the course identifying those reflected or absent from her best and worse experiences. Thus, it is important to recognize that the data collected is in part influenced by the specific teaching-learning principles that were taught in this course.

SR is a white, mid-career academic with several years of experience teaching in both online and face to face formats largely in graduate programs in the United States. SR holds three graduate degrees, two in nursing and one in physiology. The interdisciplinary training has provided an appreciation for the importance of learning experiences both in the classroom and training environment (clinical or laboratory). As a student, this experiential learning of applying what was learned in the classroom to real life situations was critical to full understanding of the material. As a DNP prepared nurse, SR has a strong interest in translating evidence into practice for the both the clinical and educational domains. This study offered an opportunity to gain a better understanding of students values and perspectives related to pedagogy, which will provide addition evidence to inform her own pedagogy. While analyzing the data, SR noted that students referenced aspects related to the affective domain frequently reinforcing her own observations that students respond positively to exercises involving self-reflection.

SSY has been teaching undergraduate nursing for four years and participation in the study was an opportunity to evaluate her own teaching strategies and recall her own experiences as an online learner. As a white professor in a school of nursing in the United States, she found herself reacting to comments from students that described how they felt they had to teach themselves the material. She recalled feeling this way herself and the frustration and anger over instructors that did not answer requests for information about assignments or give any constructive feedback for work submitted. Today as an online instructor herself, she does not want her own students to have this experience of isolation and believes effective instructors must encourage, care, connect, communicate, and remain passionate about what they are teaching. SSY feels communication is particularly important to prevent students from feeling isolated, uninformed, and that they are teaching themselves the material. As SSY coded, she noticed students valued mutual respect and this resonated with her own experiences as a student and a teacher, feeling that respect builds the trust necessary for learning.
